# Effect of triplet multiple quantum well structures on the performance of blue phosphorescent organic light-emitting diodes

**DOI:** 10.1186/1556-276X-7-23

**Published:** 2012-01-05

**Authors:** Seokjae Lee, Jaryong Koo, Gunwoo Hyung, Donghwan Lim, Donghyung Lee, Kumhee Lee, Seungsoo Yoon, Wooyoung Kim, Youngkwan Kim

**Affiliations:** 1 Department of Information Display, Hongik University, Seoul, 121-791, South Korea; 2 Department of Chemistry, Sungkyunkwan University, Suwon, 440-746, South Korea; 3 School of Display Engineering, Hoseo University, Asan, 336-795, South Korea

## Abstract

We investigate multiple quantum well [MQW] structures with charge control layers [CCLs] to produce highly efficient blue phosphorescent organic light-emitting diodes [PHOLEDs]. Four types of devices from one to four quantum wells are fabricated following the number of CCLs which are mixed p- and n-type materials, maintaining the thickness of the emitting layer [EML]. Remarkably, such PHOLED with an optimized triplet MQW structure achieves maximum luminous and external quantum efficiency values of 19.95 cd/A and 10.05%, respectively. We attribute this improvement to the efficient triplet exciton confinement effect and the suppression of triplet-triplet annihilation which occurs within each EML. It also shows a reduction in the turn-on voltage from 3.5 V (reference device) to 2.5 V by the bipolar property of the CCLs.

## Background

Due to their high efficiency, phosphorescent organic light-emitting diodes [PHOLEDs] are promising light-emitting materials in organic light-emitting diodes [OLEDs]. An internal quantum efficiency of 100% could be realized in red and green PHOLEDs [1,2]. However, the performance of blue PHOLEDs still needs to be improved for lighting applications. Light emission in PHOLEDs depends on the properties of the organic material in the devices [3,4]. In particular, the energy level of the charge transport, host, and emitter materials influences the light-emitting efficiency. Besides, many different device architectures have attempted to improve the light-emitting efficiency of PHOLEDs. Hole and electron blocking layers or triplet exciton blocking layers [TEBLs] in PHOLEDs were introduced to confine both carriers and excitons within emitting layers [EMLs] [5]. A double emitting layer structure was also employed in OLEDs by utilizing phosphorescent materials doped in two different hosts. As a result, these ways were effective in providing higher efficiency in PHOLEDs [6].

Another way to achieve high efficiency in OLEDs is to confine excitons inside the EML using the multiple quantum well [MQW] structure [7]. Only a few reports concerning the MQW structure with good carrier and exciton confinement ability have been presented on OLEDs until quite recently. For example, Qiu et al. [7] improved the charge balance by utilizing an organic MQW structure to decelerate hole transportation. Huang et al. [8] used MQW structures to increase the carrier recombination efficiency, where both charges and excitons were confined to the EMLs. Park et al. [9] and Kim et al. [10] also reported similar triplet MQW structures. Recently, Liu et al. [11] proposed a non-doping EML method, instead of a host-emitter doping method, to improve the efficient triplet exciton confinement effect and the suppression of triplet-triplet annihilation that occurs via a single-step long range (Forster-type) energy transfer between excited molecules.

In this paper, we demonstrate efficient blue PHOLEDs by using iridium(III) bis[(4, 6-di-fluorophenyl)-pyridinato-*N*,*C*^2'^]picolinate [FIrpic] doped in *N*,*N*'-dicarbazolyl-3, 5-benzene [mCP] with charge control layers [CCLs] as an MQW structure. The device architecture was developed to confine excitons inside each EML and to manage triplet excitons by controlling the charge injection. A stacked recombination zone structure, which can prevent triplet quenching processes and triplet exciton confinement within recombination region, was designed, and its performance was compared with that of blue devices. In addition, a mixed CCL, which has a p-type mCP and an n-type 2, 2',2"-(1, 3, 5-benzenetryl)tris(1-phenyl)-1H-benzimidazol [TPBi], reduced the turn-on voltage and enhanced efficiencies by prohibiting triplet exciton diffusion out of each EML.

## Methods

A glass substrate coated with a 180-nm-thick indium tin oxide [ITO] layer has a sheet resistance of 12 Ω/sq. The ITO was cleaned with acetone, methanol, distilled water, and isopropyl alcohol in an ultrasonic bath. The pre-cleaned ITO was then treated with O_2 _plasma with the conditions of 2 × 10^-2 ^Torr, 125 W, and 2 min. All organic layers were sequentially deposited onto the substrate without breaking the vacuum at a pressure of 5 × 10^-7 ^Torr, using thermal evaporation equipment. The deposition rates were 1.0 to 1.1 Å/s for organic materials and 0.1 Å/s for lithium quinolate [Liq]. Finally, the aluminum cathode was deposited at a rate of 10 Å/s. The deposition rates were controlled with a quartz crystal monitor, and the doping concentrations of the emitters were optimized. After the organic and metal depositions, the devices were encapsulated in a glove box with O_2 _and H_2_O at concentrations below 1 ppm. A desiccant material consisting of barium oxide powder was used to absorb the residue moisture and oxygen in the encapsulated device. The devices had emission areas of 3 × 3 mm. The voltage, luminance, luminous efficiency, external quantum efficiency, power efficiency, and current density characteristics were measured and immediately recorded with a Chroma meter CS-1000A (Konica Minolta Holdings, Inc., Chiyoda, Tokyo, Japan). The current and voltage were controlled with a measurement unit (model 236, Keithely Instruments Inc., Cleveland, OH, USA).

## Results and discussion

**Figure 1:**
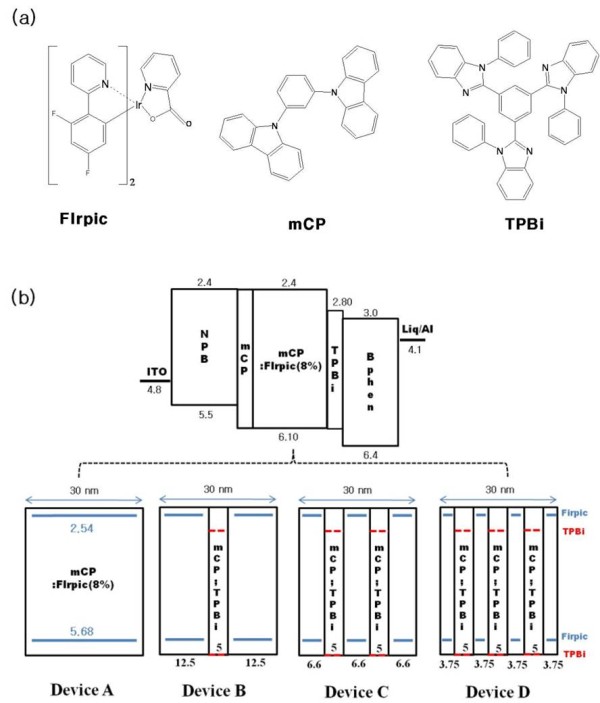
**Material and device structures**. (**a**) The chemical structures of FIrpic, mCP, and TPBi materials. (**b**) The structures and energy level diagrams of blue PHOLEDs (devices A, B, C, and D).

Figure 1a shows the chemical structures of FIrpic, mCP, and TPBi materials of blue PHOLEDs. FIrpic is the most well-known phosphorescent blue emitter, and mCP, as a carbazole-based material, is known to be a potential host material for blue electrophosphorescence because of its wide bandgap energy, high triplet energy, and good hole-transporting ability [12-14]. In addition, the high electron mobility of TPBi that provides good transport characteristics and barrier height of exciton diffusion out of EMLs do not exist due to the high triplet energy level of TPBi [15]. Figure 1b shows the structures and energy level diagrams of blue PHOLEDs (devices A, B, C, and D). A series of phosphorescent blue devices were prepared with the structure of ITO as an anode; *N*,*N*'-bis-(1-naphyl)-*N*,*N*'-diphenyl-1, 1'-biphenyl-4, 4'-diamine (NPB, 50 nm) as a hole transporting layer; mCP (5 nm) as a TEBL; single EML (device A) or MQW structure EMLs (devices B, C, and D); TPBi (10 nm) as a TEBL and electron transporting layer [ETL]; 4, 7-diphenyl-1, 10-phenanthroline (BPhen, 30 nm) as an ETL; Liq (2 nm) as an electron injection layer; and aluminum (Al, 100 nm) as a cathode. As shown in Figure 1b, devices A, B, C, and D have the following EML structure: device A, FIrpic doped in mCP (30 nm) as a reference device; device B, *n *consists of [FIrpic doped in mCP (12.5 nm)]_*n *= 2 _and [CCL (mCP/TPBi = 1:1, 5 nm)]_*n *= 1_; device C, *n *consists of [FIrpic doped in mCP (6.6 nm)]_*n *= 3 _and [CCL (mCP/TPBi = 1:1, 5 nm)]_*n *= 2_; device D, *n *consists of [FIrpic doped in mCP (3.75 nm)]_*n *= 4 _and [CCL (mCP/TPBi = 1:1, 5 nm)]_*n *= 3 _as an MQW structure device, where the doping concentrations of FIrpic were optimized at 8 wt.%, respectively. MQW structure devices with CCLs, which were a mixed mCP of hole transport type and TPBi of electron transport type, can control the carrier movement with ease.

**Figure 2:**
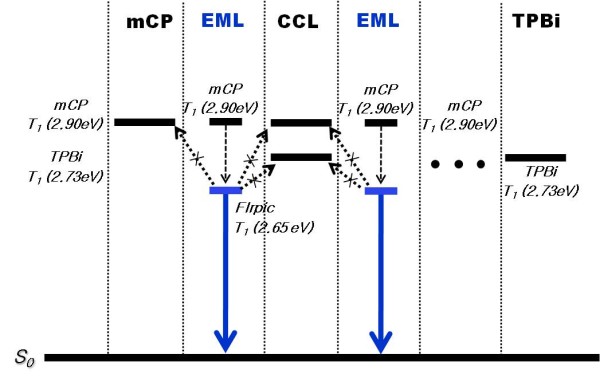
**Schematic drawing of triplet energy levels**. The lowest triplet states (*T*_1_) of materials, such as TEBL, EML, and CCL, and the triplet exciton transfer mechanism in [EML]_*n *= 2 _and [CCL]_*n *= 1 _of the triplet MQW structure. Both triplet exciton transfer and energy transition are shown in dotted arrows, while phosphorescence from the *T*_1 _state to the ground state is shown in solid arrows.

Figure 2 shows the lowest triplet states [*T*_1_] of materials, such as TEBL, EML, and CCL, and the triplet exciton transfer mechanism in [EML]_*n *= 2 _and [CCL]_*n *= 1 _of the triplet MQW structure. Both triplet exciton transfer and energy transition are shown in dotted arrows, while phosphorescence from the *T*_1 _state to the ground state is shown in solid arrows. The *T*_1 _of mCP and TPBi were 2.90 eV and 2.73 eV, respectively, compared to 2.65 eV for FIrpic [16,17]. From their triplet state alignments, it can be speculated that there should be an efficient exothermic energy transfer from the host material triplet states to the FIrpic triplet state as well as an excellent triplet energy confinement on the FIrpic molecules within each EML. It is important to suppress any back energy transfer from the emitter triplet states to the others and enable consumption of all the electrically generated triplet excitons contributing to the emission.

**Figure 3:**
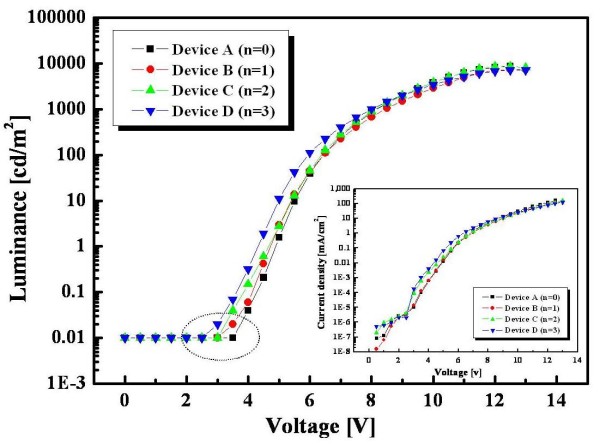
**Electrical characteristics of blue PHOLEDs**. The luminance versus voltage characteristics (inset: current density versus voltage characteristics) of devices A, B, C, and D. The dotted circle on the graph shows reduction in the turn-on voltage from 3.5 V of the reference device (device A) to 2.5 V of the MQW structure device (device D).

Figure 3 shows luminance versus voltage characteristics, and the inset of Figure 3 shows current density versus voltage characteristics of devices A, B, C, and D. The dotted circle on the graph of Figure 3 shows reduction in the turn-on voltage from 3.5 V of the reference device (device A) to 2.5 V of the MQW structure device (device D). Device A showed a lower current density than devices B, C, and D at the whole driving voltages because holes and electrons can be easily transported through the CCLs, which were a mixed p-type mCP and n-type TPBi, for controlling the carrier movement. The CCLs, including the TPBi of good electron-transporting ability, enhanced the movement of electrons within the EML and helped the holes' movement slow down compared with conventional single EML without any CCLs. In this result, the turn-on voltage of devices B, C, and D are lower than that of device A. This also indicates that devices B, C, and D are better than device A for charge balance within EMLs.

**Figure 4:**
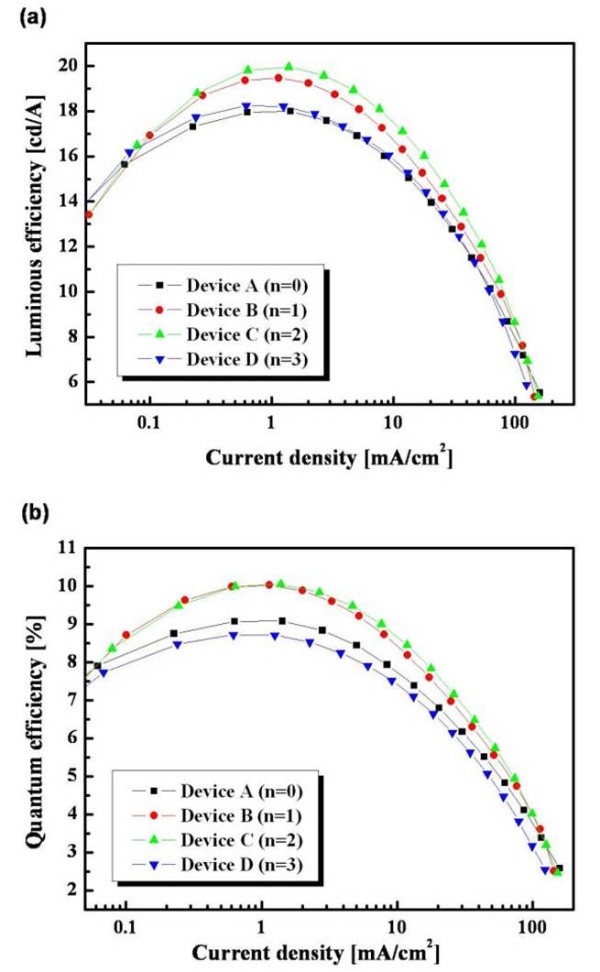
**Efficiencies of blue PHOLEDs**. (**a**) The luminous efficiency versus current density and (**b**) the external quantum efficiency versus current density of devices A, B, C, and D.

The current density, luminous efficiency, and quantum efficiency of devices A, B, C, and D were compared, as shown in Figure 4a, b. The maximum luminous efficiencies of devices A, B, C, and D were 17.99, 19.46, 19.95, and 18.24 cd/A, and the maximum quantum efficiencies of devices A, B, C, and D were 9.08%, 10.02%, 10.05%, and 8.72%, respectively. As with device C with a triplet MQW structure, *n *consists of [FIrpic doped in mCP (6.6 nm)]_*n *= 3_; [CCL (mCP/TPBi = 1:1, 5 nm)]_*n *= 2 _shows the best luminous and external quantum efficiency values of 19.95 cd/A and 10.05%, respectively. A comparison of the performance of these four devices is provided in Table 1. Device C exhibited higher efficiencies than devices A, B, and D due to the appropriate broad recombination zone by controlling the optimization of various EML thicknesses. In addition, the CCLs (mCP/TPBi) partially confine holes and electrons at the first EML, and some of the holes and electrons arrive at the other EML after transporting through the CCL. This phenomenon leads to the good charge balance and broad recombination zone for blue emission. We could control the optimization of EML thicknesses and enhance the charge balance within EMLs. As for various recombination zones, the triplet exciton diffusions are prohibited inside each EML by CCLs with a high triplet state (*T*_1_). It effectively reduces exciton quenching processes, such as triplet-triplet annihilation and triplet-polaron annihilation [18,19]. In the case of device D with a triplet MQW structure, *n *consists of [FIrpic doped in mCP (3.75 nm)]_*n *= 4_; [CCL (mCP/TPBi = 1:1, 5 nm)]_*n *= 3 _shows lower efficiencies than the other devices due to a relatively narrow emissive region.

**Table 1:** The electrical characteristics for blue PHOLEDs

Device	Current density (mA/cm^2^)	Luminance (cd/m^2^)	Turn-on voltage (V)	Luminous efficiency max. (cd/A)	Quantum efficiency max. (%)
A	115.75 (12 V)	8, 312 (12 V)	3.5	17.99	9.08
B	88.88 (12 V)	8, 521 (12 V)	3.0	19.46	10.02
C	86.56 (12 V)	7, 094 (12 V)	3.0	19.95	10.05
D	78.88 (12 V)	6, 856 (12 V)	2.5	18.24	8.72

## Conclusions

In conclusion, the present study reports on the high efficiency blue PHOLEDs based on a carrier and triplet exciton confinement inside recombination zones by using a triplet multiple quantum structure. The triplet energies of mCP and TPBi are higher than those of FIrpic. Therefore, triplet multiple quantum structures with CCLs exhibited efficient carrier and triplet exciton confinement within each EML. Moreover, CCLs can play a role in carrier movement with ease and triplet exciton blocking as expected from high triplet energy levels. In the electrical characteristic results of blue devices, the properties of device C with three recombination zones were found to be superior to the properties of devices A, B, and D. We attribute such high efficiencies and reduced turn-on voltage to the following two advantages caused by the triplet MQW structure: (1) efficient charge and exciton confinement effect by CCLs and TEBLs with high triplet energy level and (2) charge transportation balance in each EML by CCLs with bipolar property. The described MQW device concept may be useful to fabricate highly efficient devices for future OLED display and lighting applications.

## Competing interests

The authors declare that they have no competing interests.

## Authors' contributions

SL and YK conceived and designed the experiments. SL and DL carried out the experiments with contributions from GH. KL and SY designed and synthesized the materials of OLEDs. DL measured the characterization of devices. WK provided the glass substrate coated with ITO. JK supervised the work. SL and YK wrote the manuscript. All authors read and approved the final manuscript.

## References

[B1] Pfeiffer M Forrest SR Leo K Thompson ME Electrophosphorescent p-i-n organic light-emitting devices for very-high-efficiency flat-panel displays Adv Mater (Weinheim Ger) 2002 14 1633 1636 10.1002/1521-4095(20021118)14:22<1633::AID-ADMA1633>3.0.CO;2-#

[B2] Watanabe S Ide N Kido J High-efficiency green phosphorescent organic light-emitting devices with chemically doped layers Jpn J Appl Phys 2007 46 1186 1188 10.1143/JJAP.46.1186

[B3] Shen J Yang J Physical mechanisms in double-carrier trap-charge limited transport processes in organic electroluminescent devices: a numerical study J Appl Phys 1998 83 7706 7714 10.1063/1.367942

[B4] Mori T Mizutain T Application of energy transfer model to partially DCM-doped Alq_3 _light-emitting diode Polym Asv Technol 1997 8 471 476 10.1002/(SICI)1099-1581(199707)8:7<471::AID-PAT675>3.0.CO;2-G

[B5] Adamovich V Cordero SR Djurovich PI Tamayo A Thompson ME Andrade B Forrest SR New charge-carrier blocking materials for high efficiency OLEDs Org Electron 2003 4 77 87 10.1016/j.orgel.2003.08.003

[B6] Lee JH Lee JJ Chu HY Investigation of double emissive layer structures on phosphorescent blue organic light-emitting diodes Synth Met 2009 159 1460 1463 10.1016/j.synthmet.2009.03.026

[B7] Qui Y Gao Y Wei P Wang L Organic light-emitting diodes with improved hole-electron balance by using copper phthalocyanine/aromatic diamine multiple quantum wells Appl Phys Lett 2002 80 2628 2630 10.1063/1.1468894

[B8] Huang JS Yang KX Liu SY Jiang HJ High-brightness organic double-quantum-well electroluminescent devices Appl Phys Lett 2000 77 1750 1752 10.1063/1.1311313

[B9] Park TJ Jeon WS Choi JW Pode R Jang J Kwan JH Efficient multiple triplet quantum well structures in organic light-emitting devices Appl Phys Lett 2009 95 103303 10.1063/1.3224190

[B10] Kim SH Jang J Hong JM Lee JY High efficiency phosphorescent organic light emitting diodes using triplet quantum well structure Appl Phys Lett 2007 90 173501 10.1063/1.2731435

[B11] Divayana Y Sun XW Observation of excitonic quenching by long-range dipole-dipole interaction in sequentially doped organic phosphorescent host-guest system Phys Rev Lett 2007 99 143003 17930667 10.1103/PhysRevLett.99.143003

[B12] Holmes RJ Forrest SR Tung YJ Kwong RC Brown JJ Garon S Thompson ME Blue organic electrophosphorescence using exothermic host-guest energy transfer Appl Phys Lett 2003 82 2422 2424 10.1063/1.1568146

[B13] Tokito Iijima T Suzuri Y Kita H Tsuzuki T Sato F Confinement of triplet energy on phosphorescent molecules for highly-efficient organic blue-light-emitting devices Appl Phys Lett 2003 83 569 571 10.1063/1.1594834

[B14] Lei GT Wang LD Duan L Wang JH Qiu Y Highly efficient blue electrophosphorescent devices with a novel host material Synth Met 2004 144 249 252 10.1016/j.synthmet.2004.03.010

[B15] Hung WY Ke TH Lin YT Wu CC Hung TH Chao TC Wong KT Wu CI Employing ambipolar oligofluorene as the charge-generation layer in time-of-flight mobility measurements of organic thin films Appl Phys Lett 2006 88 064102 10.1063/1.2172708

[B16] Seo JH Seo JH Park JH Kim JH Hyung GW Lee KH Yoon SS Kim YK Highly efficient white organic light-emitting diodes using two emitting materials for three primary colors (red, green, and blue) Appl Phys Lett 2007 90 203507 10.1063/1.2740191

[B17] Ren X Li J Holmes RJ Djurovich PI Forrest SR Thompson ME Ultrahigh energy gap hosts in deep blue organic electrophosphorescent devices Chem Mater 2004 16 4743 4747 10.1021/cm049402m

[B18] Baldo MA Adachi C Forrest SR Transient analysis of organic electrophosphorescence. II. Transient analysis of triplet-triplet annihilation Phys Rev 2000 62 10967 10977 10.1103/PhysRevB.62.10967

[B19] Reineke S Walzer K Leo K Triplet-exciton quenching in organic phosphorescent light-emitting diodes with Ir-based emitters Phys Rev 2007 75 125328

